# BTEB2 Prevents Neuronal Apoptosis via Promoting Bad Phosphorylation in Rat Intracerebral Hemorrhage Model

**DOI:** 10.1007/s12031-014-0305-8

**Published:** 2014-04-27

**Authors:** Xiaojuan Liu, Damin Yuan, Xiaoke Nie, Jianhong Shen, Yaohua Yan, Dongmei Zhang, Jianxin Gu

**Affiliations:** 1 Key Laboratory of Glycoconjugate Research, Ministry of Health, Department of Biochemistry and Molecular Biology, Shanghai Medical College, Fudan University, Shanghai, 200032 China; 2 Department of Pathogen Biology, Medical College, Nantong University, Nantong, Jiangsu 226001 China; 3 Department of Nutrition and Food Hygiene, School of Public Health, Nantong University, Nantong, Jiangsu 226001 China; 4 Department of Neurology, Affiliated Hospital of Nantong, Nantong University, Nantong, Jiangsu 226001 China; 5 Medical College, Nantong University, Nantong, Jiangsu 226001 China

**Keywords:** Intracerebral hemorrhage, BTEB2, Neuron, Apoptosis

## Abstract

Krüppel-like zinc-finger transcription factor 5 (KLF5), known as BTEB2 or IKLF, has several biological functions that involve cell proliferation, development and apoptosis. Previous studies demonstrated that BTEB2 had anti-apoptotic effect in multiple diseases such as esophageal cancer and non-small cell lung cancers (NSCLCs). However, the distribution and function of BTEB2 in CNS diseases remain unknown. In this study, we show that BTEB2 down-regulates neuronal apoptosis during pathophysiological processes of intracerebral hemorrhage (ICH). A rat ICH model was established by behavioral tests. Western blot and immunohistochemistry revealed a remarkable up-regulation of BTEB2 expression surrounding the hematoma after ICH. Double-labeled immunofluorescence showed BTEB2 was mostly co-localized with neurons, rarely with activated astrocytes and microglia. Furthermore, we detected that neuronal apoptosis marker active caspase-3 had co-localizations with BTEB2. In addition, KLF5 knockdown in vitro specifically resulted in increasing neuronal apoptosis coupled with reduced Bad phosphorylation at both ser112 and ser136 residues. All our findings suggested that BTEB2 down-regulated neuronal apoptosis via promoting Bad phosphorylation after ICH.

**Electronic supplementary material:**

The online version of this article (doi:10.1007/s12031-014-0305-8) contains supplementary material, which is available to authorized users.

## Introduction

Intracerebral hemorrhage (ICH) occurs when a diseased blood vessel ruptures, allowing blood to leak into the surrounding brain (Rui et al. 2013). Spontaneous ICH accounts for a significant proportion of all strokes and is one of the leading causes of stroke related mortality and morbidity worldwide (Sun et al. 2013). Although a lot of resources have been thrown into clinical and basic research regarding ICH, the prognosis of patients is still very poor. It is an urgent work to improve the clinical outcome of ICH, investigation into the pathogenesis of ICH-induced brain injury. The primary and secondary damage both contribute to the pathological processes following ICH (Aronowski and Zhao 2011). Apart from the primary damage caused by the mass effect due to hematoma formation, subsequent parallel pathological pathways all lead to the secondary damage. Cytotoxicity of blood, oxidative stress and neuronal apoptosis are the most crucial event consists of perplexing pro-apoptotic activations and subtle anti-apoptotic accommodation. Classical apoptosis is usually initiated by two main pathways: the intrinsic and the extrinsic pathways (Aronowski and Zhao 2011). The activation of extrinsic pathway is triggered by ligands binding with their respective death receptors. Caspase-3 has also been proved to be a frequently activated death protease in mammalian cell apoptosis (Gong et al. 2001).

Krüppel-like zinc-finger transcription factor 5 (KLF5), also named BTEB2 or IKLF, belongs to the KLF family (Taniguchi Ishikawa et al. 2013). Members of this family are made up of over 20 identified members. They are characterized by possessing a highly conserved C-terminal and three zinc finger DNA-binding domains that bind to GC-rich sequences (Dang et al. 2002; Philipsen and Suske 1999; Shi et al. 1999; Turner and Crossley 1999). Human KLF5 is a member of the mammalian Krüppel-like transcription factors, found to be dynamically regulated at the transcription level in mouse (Shi et al. 1999). KLF5 is widely expressed at varying levels in different tissues such as colon, small intestine (Chen et al. 2002; Conkright et al. 1999; Shi et al. 1999). KLF5 has recently attracted attention because of its important regulatory activities related to diverse functions such as cell growth, proliferation and tumorigenesis (Suzuki et al. 2007). As a basic transcription factor, KLF5 regulates a good number of important target genes associated with cell cycle such as cyclinD1, cyclinB, PDGFα and FGF-BP. KLF5 also has essential roles in cell apoptosis, migration and differentiation (Zhao et al. 2008). Mechanical induction of KLF5 can result in direct transactivation of cyclinD1 expression, which subsequently stimulates cell proliferation (Suzuki et al. 2009). Furthermore, KLF5 attenuates cleaved caspase-3 expression under conditions of apoptotic stimulation. KLF5 functions to mediate vascular remodeling via direct and specific stimulation of cell growth and by inhibiting apoptosis (Nakajima et al. 2012). Y Zhao and his colleagues found that KLF5 suppressed p53-independent apoptosis through Pim1 survival kinase in HCT116 p53−/−cells (Zhao et al. 2008). Recent data have shown that apoptosis can be controlled by multiple of survival pathways, which converge to promote Bad phosphorylation (Bergmann 2002). The participation of BTEB2 in various cellular activities such as apoptosis and proliferation has drawn increasingly attention (Dong and Chen 2009; Tarapore et al. 2013; Courboulin et al. 2011). However, our understanding of the specific roles of BTEB2 in adult brain after ICH remains unclear.

In this report, we show that BTEB2 attenuates neuronal apoptosis following ICH by in vivo and in vitro experiments. We for the first time investigated the expression and distribution of BTEB2 in rat caudate putamen around the hematoma after ICH.

## Materials and Methods

### Animals and the ICH Model

All animal care and surgical interventions were implemented according to the National Institutes of Health Guidelines for the Care and Use of Laboratory Animals (National Research Council 1996, USA); all animal procedures were acknowledged by the Department of Animal Center, Medical College of Nantong University. Male Sprague–Dawley rats with an average weight of 250 g were used. The rats were anesthetized intraperitoneally with sodium pentobarbital (50 mg/kg) and then positioned in a stereotaxic frame. A cranial burr hole was drilled near the right coronal suture 3.5 mm lateral t*o* the midline (Sun et al. 2013). Autologous whole blood (50 μl) was collected in a sterile syringe by cutting the tail tip (Xue and Del Bigio 2003). The sterile syringe was inserted stereotaxically into the right caudate putamen (coordinates: 0.2 mm anterior, 5.5 mm ventral, 3.5 mm lateral to the bregma) (Xue and Del Bigio 2003). The sham group only had a needle insertion. The needle was removed 10 min after injection, the skin incision closed and the animals were allowed to recover. Experimental animals (*n* = 6–8 per time point) were killed to extract the protein for Western blot analysis at 6 h, 12 h, 1 day, 2 days, 3 days, 5 days, 7 days and 9 days after ICH. Rats in the sham group (*n* = 3) were sacrificed on the third day. An additional two rats at each time point from the experimental group were killed for pathologic studies. All efforts were made to minimize the number of animals used and their suffering.

### Exclusions and Mortality

Three animals in Western blot, five in immunohistochemistry and four in immunofluorescent were excluded due to technical difficulties, experimenter error and mortality. Mortality occurred only after the operation error to cause severe ICH. In total, seven animals had a severe ICH, four of which died within 24 h (*p* = 0.215 vs. SHAM).Three died quickly after ICH. After exclusions, 12 rats remained (ICH: *n* = 6, SHAM: *n* = 6), immunohistochemistry had ten rats (ICH: *n* = 6, SHAM: *n* = 4) and immunofluorescent had 16 rats (ICH: *n* = 8, SHAM: *n* = 8).

### Cell Cultures and Stimulation

PC12 cells were cultured in Dulbecco’s modified medium with 10 % (v/v) fetal bovine serum, 5 % donor horse serum and antibiotics at 37 °C under 5 % CO_2_ in humidified air. The cells were passed every 3–4 days. To study apoptosis, cells were seeded onto a poly-l-lysine-coated 60-mm dishes and incubated in a low concentration of serum (1 % horse serum) for 24 h prior to treatment with hemin in 100 μmol/l at different time points.

### Forelimb Placing Test

Held the rats by torsos, thus the forelimb could hang free. Independent testing of each forelimb was elicited by brushing the respective vibrissae on the corner edge of a countertop. Intact rats lay up the forelimb quickly onto the countertop. According to the extent of injury, placing of the forelimb contralateral to the injury may be impaired. During the experiments, each rat was tested ten times for each forelimb and the percentage of trials in which the rat placed the left forelimb was calculated (Karabiyikoglu et al. 2004).

### Corner Turn Test

The rats were allowed to proceed into a corner, the angle of which was 30°. To exit the corner, the rat should turn either to the left or to the right. Only the turns involving full rearing along either wall was included (a total of eight per animal). According to the extent of injury, rats may show a tendency to turn to the side of the injury. The percentage of right turns was used as the corner turn score. The rats were not picked up instantly after each turn so that they would not develop an aversion for their prepotent turning response (Hua et al. 2002).

### Western Blot Analysis

Rats were sacrificed at different time points after operation once given an overdose of chloral hydrate. The caudate putamen tissues surrounding the hematoma (2 mm from the incision) and the equal part of the contralateral or sham group were excised and instantly frozen at −80 °C until use. To prepare lysates, frozen brain tissue samples were minced with eye scissors in ice. The samples were then homogenized in lysis buffer (1 % NP-40, 50 mmol/l Tris, pH = 7.5, 5 mmol/l EDTA, 1 % SDS, 1 % sodium deoxycholate, 1 % Triton X-100, 1 mmol/l PMSF, 101 g/ml aprotinin, 11 g/ml leupeptin) and clarified by centrifuging for 20 min in a microcentrifuge at 4 °C. After the determination of concentration with the Bradford assay (Bio-Rad), the samples were subjected to SDS-polyacrylamide gel electrophoresis and transferred to a polyvinylidene diflouride (PVDF) filter membrane by a transfer apparatus at 350 mA for 2 h. The membrane was then blocked with 5 % nonfat milk and incubated with primary antibody against BTEB2 (anti-rabbit, 1:1,000; Santa Cruz), active caspase-3 (anti-mouse, 1:1,000; Cell Signaling), GAPDH (anti-rabbit, 1:1,000; Sigma) at 4 °C overnight. Lastly, the membrane was incubated with a second antibody for 2 h and visualized using an enhanced chemiluminescence system (Pierce Company, USA).

### Sections and Immunohistochemistry

After determined survival time points, the rats were deeply anesthetized with chloral hydrate (10 % solution) and perfused pericardially with 500 ml 0.9 % saline followed by 4 % paraformaldehyde. After perfusion, the brains were removed and post-fixed in the same fixative for 3 h and then replaced with 20 % sucrose for 2–3 days, followed by 30 % sucrose for 2–3 days. The tissues were embedded in OCT compound when needed. Then, 6–7 μm frozen cross-sections were prepared and examined. All sections were stored at −20 °C before use. After the sections had been prepared, they were kept in an oven at 37 °C for 30 min and rinsed twice in 0.01 M PBS (pH 7.2) for 5 min. Then the sections were blocked with confining liquid consisting of 10 % donkey serum, 1 % BSA, 0.3 % Triton X-100 and 0.15 % Tween-20 for 2 h at room temperature, then incubated with anti-BTEB2 antibody (Rabbit, 1:100, Santa Cruz) overnight at 4 °C, followed by incubation in biotinylated secondary antibody (Vector Laboratories, Burlingame, CA, USA). Staining was visualized with DAB (Vector Laboratories). After reactions, the sections were dehydrated, cleared and coverslipped. We examined the sections and counted the cells with strong or moderate brown staining, weak or no staining as positive or negative BTEB2 cells respectively from each group at higher magnification with a Leica light microscope (Leica, DM 5000B; Germany).

### Double Immunofluorescent Staining

For double immunofluorescent labeling, sections were prepared as previously mentioned. After air-dried for 1 h, sections were first blocked with solution containing 10 % normal serum (same species as the secondary antibody), 3 % (w/v) BSA, 0.1 % Triton X-100 and 0.05 % Tween-20 for 2 h at room temperature. Then the slides were incubated overnight with primary antibody anti-BTEB2 (rabbit, 1:100; Abcam) and different markers as follows: NeuN (mouse; 1:100; Chemicon), GFAP (mouse; 1:100; Sigma), CD11b (mouse; 1:50; Serotec), active caspase-3 (mouse or rabbit; 1:200; Santa Cruz), at 4 °C. In brief, sections were incubated with both primary antibodies overnight at 4 °C and a mixture of FITC- and TRITC-conjugated secondary antibodies (Jackson ImmunoResearch) for 2 h at 4 °C. In order to detect the morphology of the nucleus, sections were covered with DAPI (0.1 mg/ml in PBS; Sigma) for 1 h at 30 °C. The stained sections were examined with a Leica fluorescence microscope (Leica, DM 5000B; Germany).

### Quantitative Analysis

Cells double labeled for BTEB2 and cell phenotypic markers used in the experiment were quantified. Sections were double labeled with NeuN, GFAP, CD11b and active caspase-3. To identify the ratio of each phenotype-specific marker-positive cells expressing BTEB2, at least 200 phenotype-specific marker-positive cells were counted 3 mm surrounding the hematoma in each section. Then, double-labeled cells for BTEB2 and phenotype-specific markers were recorded. A total of three adjacent sections per animal were sampled.

### BTEB2 siRNA and Transfection

Double-stranded siRNA of 19 nucleotides were synthesized by Genechem. The targeting sequence of rat BTEB2 mRNA (5′-gccccggttaatttgcatat-3′, 5′-cccagtatcagcagaaggacat-3′), corresponds to the region 2676–2695, 3104–3125, respectively, relative to the first nucleotide of the start codon (GenBank Accession No. NM_001730). Non-silencing control siRNA is an irrelevant siRNA with random nucleotides and no known specificity. Transfection of PC12 cells with siRNA was performed using lipofectamine 2000 reagents (Invitrogen) according to the manufacturer’s instructions. For mock transfection, all procedures listed above were performed in the absence of siRNA. For transient transfection, the BTEB2 expression vector, the non-specific vector were carried out using lipofectamine 2000 (Invitrogen) and plus reagent in OptiMEM (Invitrogen) as suggested by the manufacturer. Transfected cells were used for the subsequent experiments 48 h after transfection.

### Statistical Analysis

All data were analyzed with Stata 7.0 statistics software. Values were expressed as mean ± SEM. The statistical significance of differences between groups was determined by one-way analysis of variance followed by Tukey’s post hoc multiple comparison tests. A *p* value <0.05 was considered statistically significant. Each experiment consisted of at least three replicates per condition.

## Results

### Rat ICH Model Was Established by Behavioral Tests

**Fig. 1:**
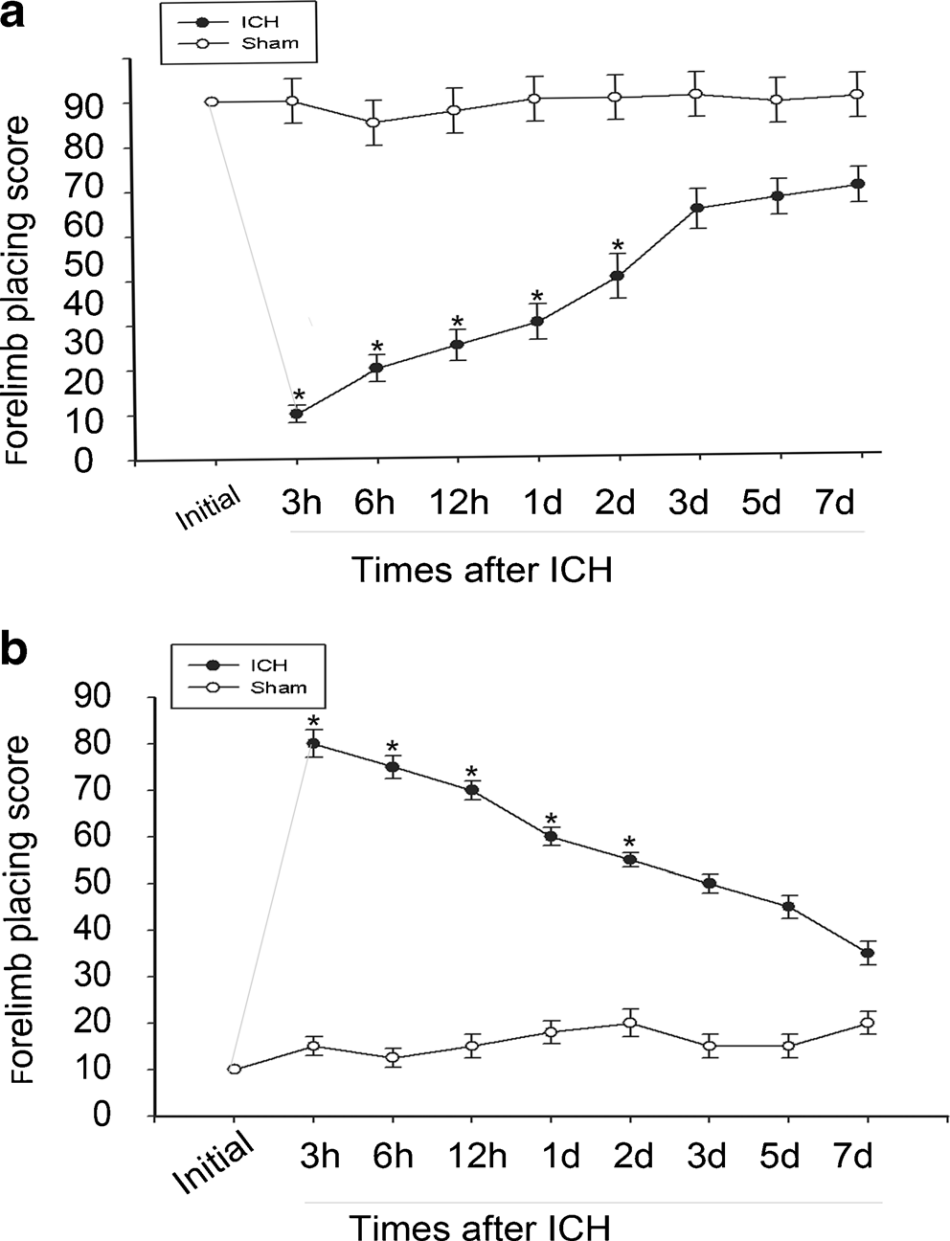
Assessments and scores of behavioral tests on rats after ICH. Behavioral tests were implemented in rats after ICH or sham operation. Forelimb placing test (**a**) and Corner turn test (**b**) scores at different survival times after ICH. The ICH group showed distinctly deficits compared with the sham group over the first 3 days (*p < 0.05) with no significant difference at baseline or 3 days later

Rats in the sham and ICH groups were underwent forelimb placing and corner turn test at different survival time points, respectively. The ICH group was distinctly worse impaired than the sham group in the first 5 days after ICH (*p < 0.05). By 7 days and thereafter, however, all rats returned to normal behavioral levels (Fig. 1a, b).

### BTEB2 Protein Expression After ICH

**Fig. 2:**
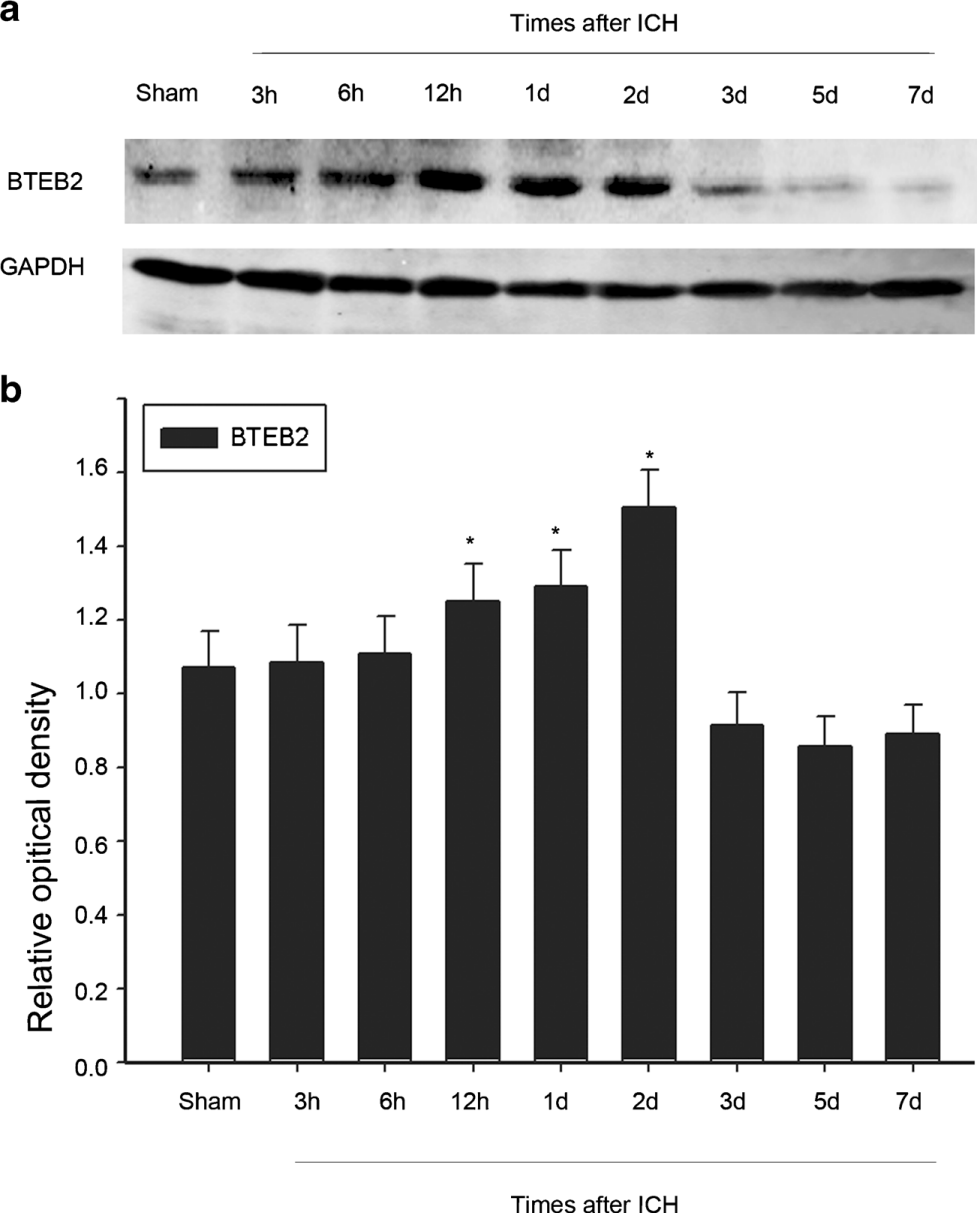
BTEB2 protein expression following ICH. Western blot was performed to study the protein level of BTEB2 surrounding the hematoma at different survival times (**a**). Quantification graphs of the intensity of staining of BTEB2 to GAPDH at each time point (**b**). Data are presented as mean ± SEM (*n* = 3, *p < 0.05)

To quantify BTEB2 expression profile after ICH, we measured the BTEB2 protein expression using Western blot analysis at different time points. The BTEB2 protein level was relatively low in the sham group, whereas it increased at 6 h after ICH, reached the peak at day 2, then gradually decreased in the perihematomal region (Fig. 2a, b). On the other hand, we also detected BTEB2 protein expression in the contralateral brain, but failed to find any significant fluctuations (data not shown). The above data indicated that BTEB2 protein level had a temporal change caused by ICH, suggesting that BTEB2 maybe played a role during ICH pathological process.

### Expression and Distribution of BTEB2 by Immunohistochemistry

**Fig. 3:**
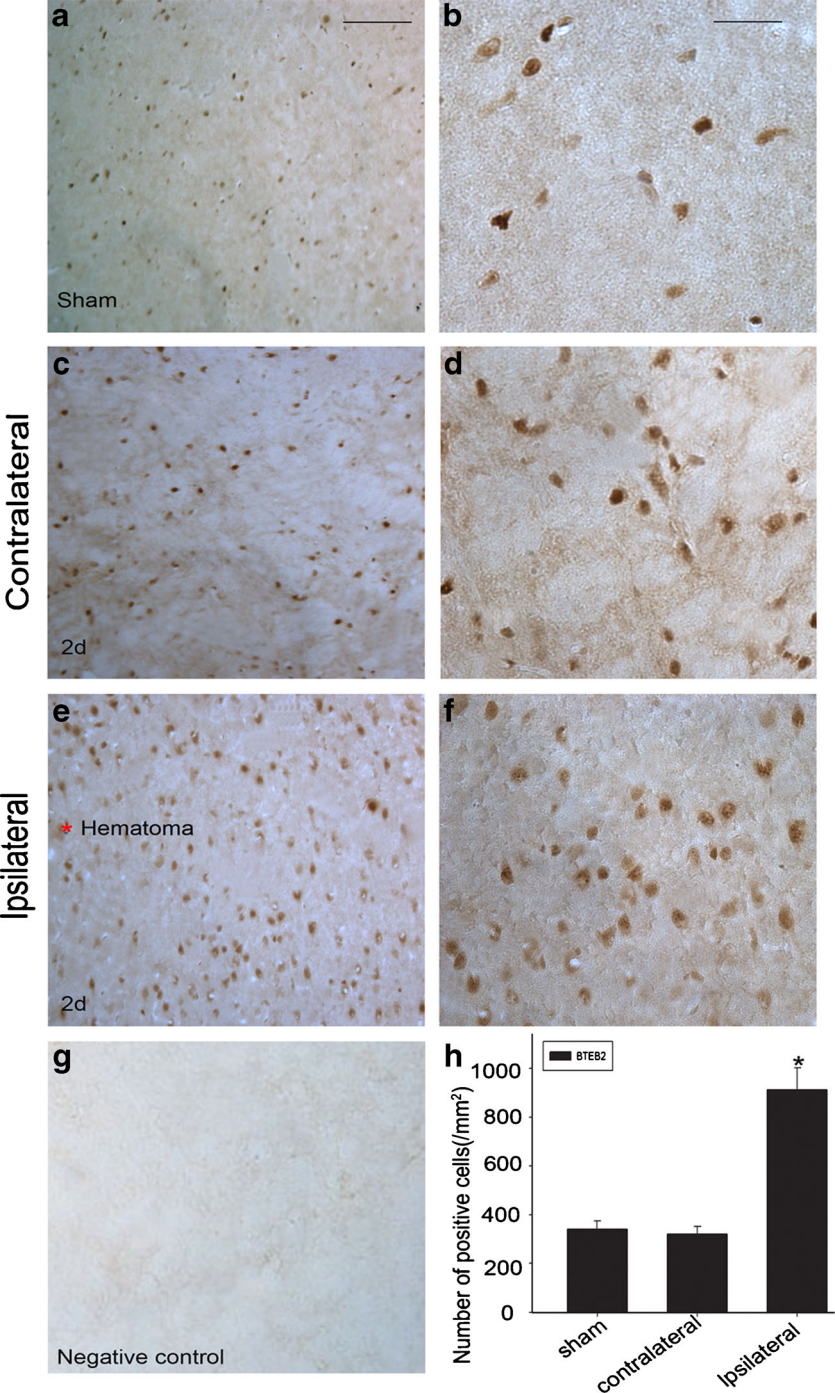
Representative microphotographs for BTEB2 immunohistochemistry surrounding the hematoma. In the sham group, BTEB2 was low (**a**, **b**). At 2 days after ICH, comparison of BTEB2 between contralateral (**c**, **d**) and ipsilateral brain caudate putamen (**e**, **f**), many cells around the hematoma showed BTEB2, the positively stained intensity (**e**, **f**). No positive signals were found in the negative control (**g**). The number of BTEB2 cells was largely increased comparing the ipsilateral group with the sham and contralateral groups (**h**). *p < 0.05. Scale bar: *left column*, 50 μm; *right columns*, 10 μm

To determine the distribution of BTEB2 in brain after ICH, we carried out immunohistochemistry on transverse cryosections of brains. BTEB2 expression dramatically increased in the brain tissue surrounding the hematoma at day 2 after ICH (Fig. 3e, f, h). In the sham and contralateral brains, BTEB2 expression was weak (Fig. 3a, b, c, d). No staining signal was observed in the negative control (Fig. 3g). These results implied that the temporal pattern of BTEB2 after ICH was consistent with the results of Western blot.

### BTEB2 Co-localized with Neurons After ICH

**Fig. 4:**
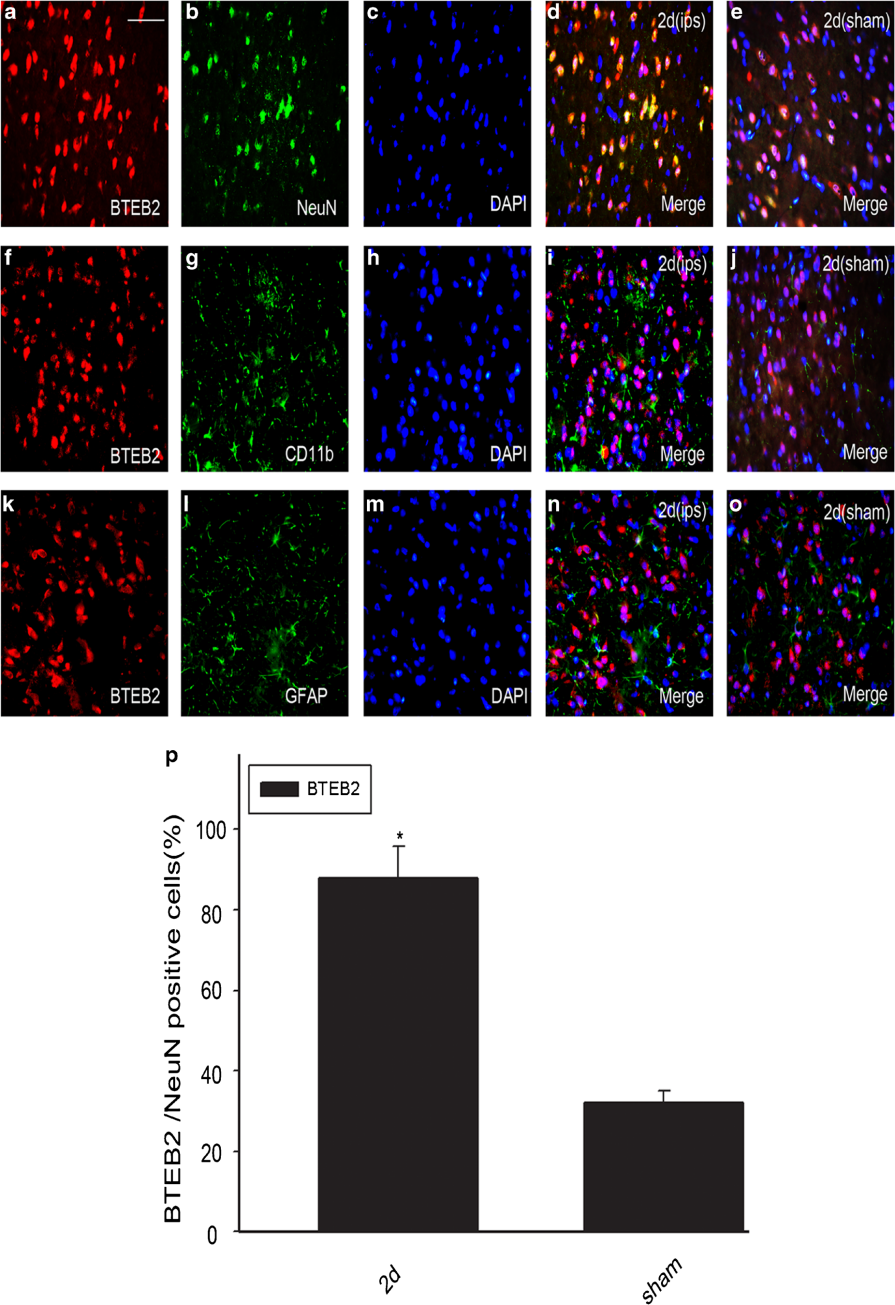
Double immunofluorescence staining for BTEB2 with different phenotype-specific markers in brain caudate putamen surrounding the hematoma. In the adult rat caudate within 3-mm distance from the hematoma at the second day after ICH, horizontal sections were labeled with BTEB2 (*red* **a**, **f**, **k**), different cell markers (*green* **b**, **g**, **l**) such as neuronal marker (NeuN), microglia marker (CD11b) and astrocyte marker (GFAP) and as well as DAPI (*blue* **c**, **h**, **m**) to show the nucleus. The *yellow and white color* visualized in the merged images represents the colocalization of BTEB2 with different phenotype-specific markers (**d**, **e**) and the **purple** indicates the colocalization of the nucleus with phenotype-specific markers (**d**, **e**, **i**, **j**, **n**, **o**). Colocalization of BTEB2 with different phenotype-specific markers in the sham group (**e**, **j**, **o**) are shown in the caudate. Quantitative analysis of NeuN-positive cells expressed BTEB2 (%) in the sham group and 2 days after ICH. (*ipsi*) indicates the perihematomal region and (*sham*) presents the sham group. *Significant difference at *p* < 0.05 compared with the sham group (**p**). *Error bars* represent SEM. Scale bars, 20 μm (**a**)

In order to further study BTEB2 cellular localization in the brain after ICH, double labeling immunofluorescent was performed in transverse cryosections of brain tissues with cell-specific markers: NeuN (a marker of neuron), GFAP (a marker of astrocyte), CD11b (a marker of microglia), to distinguish different cell type. According to the above Western blot and immunohistochemistry results, the sham and ipsilateral rat brain tissues at 2 days after ICH were selected for detection. We found that BTEB2 co-located with neurons in the cytoplasma and nucleus (Fig. 4a–e). Besides, the positive signals in the peri-ICH region were more than those in the sham group. However, no significant changes of BTEB2 expression were observed in astrocytes, and microglia between sham and ICH group (Fig. 4f–o). We also performed the DAPI labeling and detected the nuclear condensation in neurons at day 2 after ICH (Fig. 4c, h, m).

To understand the ratio of neurons expressing BTEB2, cell counting was performed between ICH 2 day group and the sham group. After ICH, BTEB2 expression significantly increased merely in neurons, compared with that of the sham group (Fig. 4p). These results showed that BTEB2 localized in the neuronal cytoplasm and nucleus.

### BTEB2 Associated with Neuronal Apoptosis After ICH

**Fig. 5:**
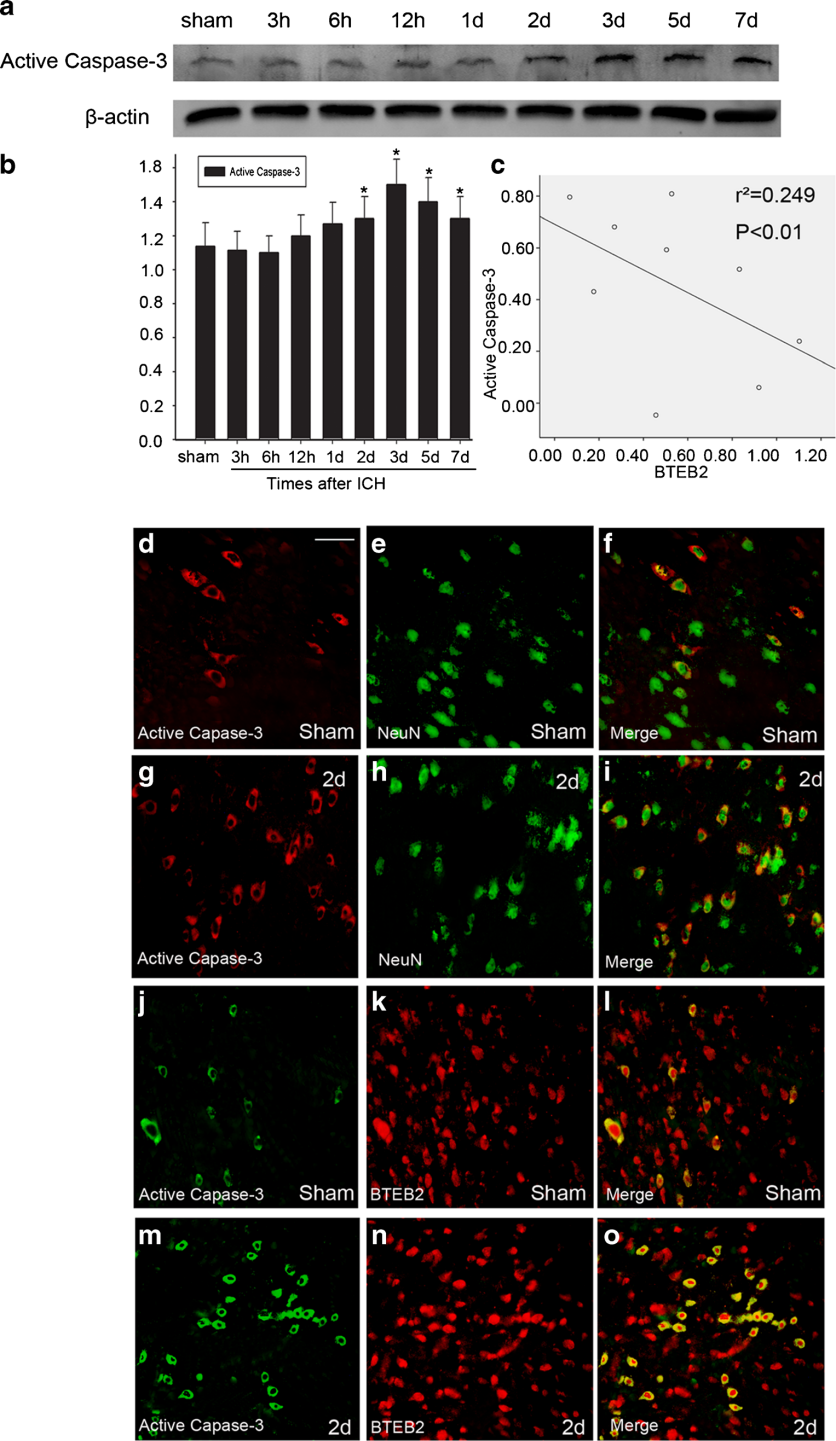
Association of BTEB2 with neuronal expressed active caspase-3 after ICH. The expression of active caspase-3 increased and peaked at day 3 after ICH, GAPDH was used to confirm that equal amount of protein was run on gel (**a**).Quantification graphs (relative optical density) of the intensity of staining of active caspase-3 and GAPDH at each time points (**b**). Negative correlation between BTEB2 protein expression and active caspase-3 protein expression after ICH (**c**). The data are mean ± SEM (*p* < 0.05, *asterisk* [*] indicates statistical significance from the sham group). Sections labeled with active caspase-3 (**d**, **g**, **j**, **m**), NeuN (**e**, **h**), BTEB2 (**k**, **n**), and the co-localization of active caspase-3/NeuN and active caspase-3/BTEB2 surrounding the hematoma at day 2 after ICH (**f**, **i**, **l**, **o**). Scale bars, 20 μm (**d–o**)

Previous studies have provided the evidence for neuronal apoptosis surrounding the hematoma after ICH (Gong et al. 2001) and also showed that BTEB2 associated with cell apoptosis (Dong et al. 2013). Thus, Western blot was used to detect active caspase-3 protein expression and immunofluorescent was applied to test its co-localizations with BTEB2. The active-caspase-3 protein expression was steady before day 2 after ICH, then increased and peaked at day 3 (Fig. 5a, b), showing opposite tendency of BTEB2 protein expression. In other words, there was negative correlation between BTEB2 protein expression and active caspase-3 protein expression after ICH (Figs. 2a, b and 5c). Furthermore, the colocalizations of BTEB2/active caspase-3 in addition to active caspase-3/NeuN (Fig. 5d–o) indicated BTEB2 might participate in neuronal apoptosis after ICH.

### BTEB2 Regulates Neuronal Apoptosis and Bad Phosphorylation In Vitro

**Fig. 6:**
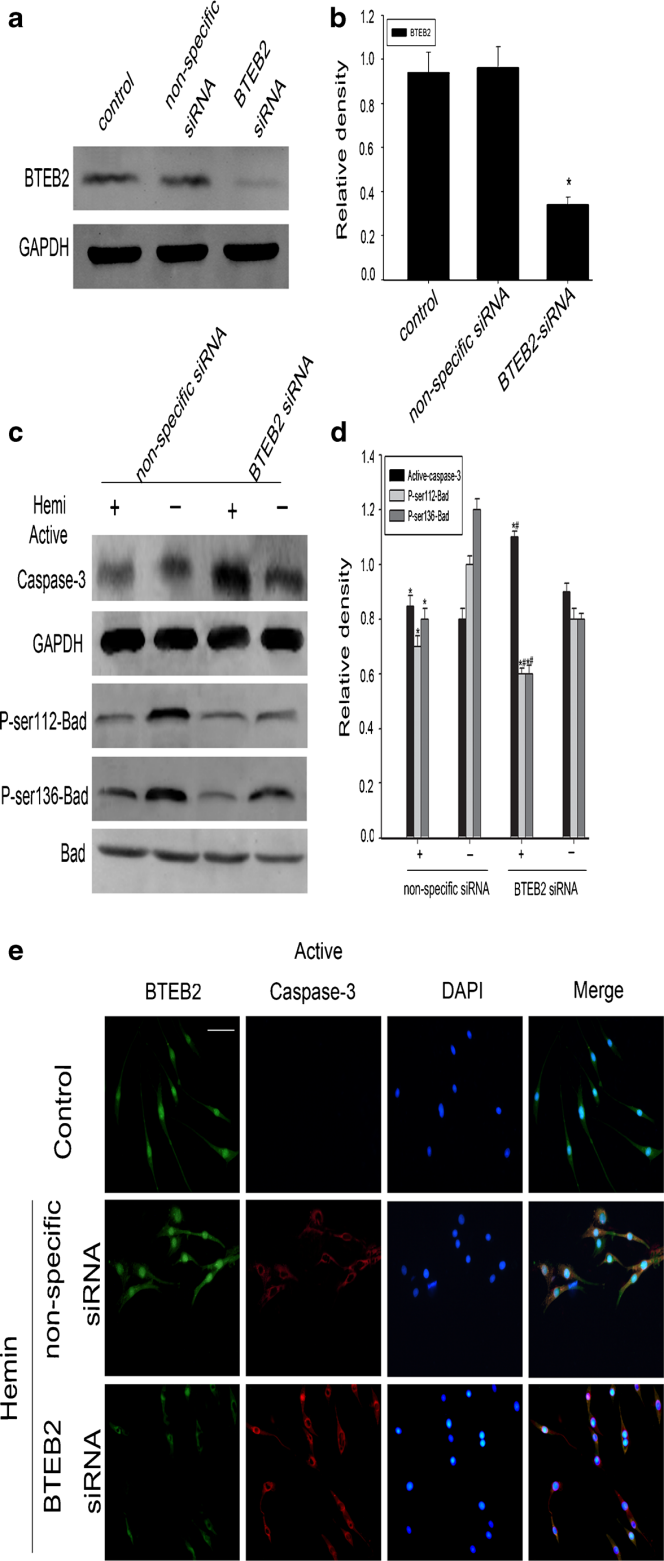
BTEB2 prevents neuronal apoptosis through Bad phosphorylation. Western blot showed siRNA knocked down BTEB2 expression in PC12 cells (**a**). Knocking down BTEB2 induced increasing levels of active caspase-3 and reduced the levels of P-ser112-bad and P-ser136-bad in hemin-treated PC12 cells (**c**). The *bar chart* indicates the density of active caspase-3/BTEB2/P-ser112-Bad/P-ser136-Bad versus GAPDH (**b**, **d**). Data are presented as means ± SEM (*p < 0.05, ^#^ *p* < 0.05). Immunofluorescence showed the co-localization of BTEB2/active-caspase-3 in control and non-specific siRNA or BTEB2 siRNA transfected PC12 cells after hemin stimulus; meanwhile, the phenotype changes of nuclei were also investigated via DAPI staining (**e**)

To identify the exact role of BTEB2 in neuronal apoptosis after ICH, BTEB2 siRNA was employed to knockdown BTEB2 protein expression in PC12 cells in vitro. BTEB2 siRNA markedly knocked down BTEB2 protein expression (Fig. 6a, b). The knockdown of BTEB2 can increase active caspase-3 expression and also specifically resulted in reduced Bad phosphorylation at both ser112 and ser136 residues with or without hemin stimulation (Fig. 6c, d). Furthermore, cell immunofluorescent staining displayed the co-localization of BTEB2 and active caspase-3 in PC12 cells (Fig. 6e).These experiments demonstrated that BTEB2 restrain neuronal apoptosis through Bad phosphorylation pathway.

## Discussion

ICH is a common fatal stroke which kills about 400,000 people annually in China. Even if the patient survives the disease, the resulting hematoma within brain parenchyma causing a series of adverse events triggers secondary insults and severe neurological deficits (Aronowski and Zhao 2011). The present study built an ICH rat model to imitate clinical ICH by performed a controlled autologous blood injection ICH model in adult rats. Our ICH model rats showed obvious behavioral performance deficits. BTEB2 was rapidly up-regulated in the perihematomal region after ICH. Double label immunofluorescence suggested that BTEB2 mostly co-located with neurons, but few astrocytes or microglia. Furthermore, there was a concomitant up-regulation of active caspase-3 with that of BTEB2 in a time-dependent manner. In vitro, knockdown of BTEB2 promoted neuronal apoptosis via inhibiting Bad phosphorylation. In conclusion, BTEB2 down-regulates neuronal apoptosis via enhancing Bad phosphorylation after ICH.

Except for the primary damage, ICH lead to series of unfavorable events, which cause following secondary damage and finally give rise to serious neurological deficits or even death (Aronowski and Zhao 2011). Neuronal apoptosis, astrocytic proliferation and oligodendrocytic death all occurred in these processes. Among them, neuronal apoptosis is the most severe consequence after ICH (Sun et al. 2013). Apoptosis, also known as programmed cell death, includes both extrinsic and intrinsic pathways. There are some overlaps between the two pathways and they both activate caspase-3 in the end. Caspase-3 activation involved neuronal apoptosis after ICH. Active caspase-3 expression is remarkably increased after ICH (Rui et al. 2013). The secondary injury following ICH comprises both anti-apoptotic and pro-apoptotic signaling cascades. Pro-apoptotic agents including p53, Bax and caspase-3 are increased in rat ICH models (Ke et al. 2013). BTEB2 expression increase in neurons in our study suggests that BTEB2 may play a role during neuronal apoptosis after ICH. Our result showed that active caspase-3 was up-regulated after injection, peaked at day 3. Not surprisingly, active caspase-3 expression showed opposite tendency to that of BTEB2, indicating that BTEB2 may downregulate neuronal apoptosis after ICH.

The role of Bad in apoptotic control is undisputed (Chao and Korsmeyer 1998). More importantly, the control of Bad activity appears to be at the junction where various signal transduction pathways are coupled to apoptosis (Horne et al. 2014). When unphosphorylated, active Bad induces apoptosis by sequestering anti-apoptotic members of the Bcl2 family (e.g., Bcl2 and BclXL), permitting aggregation of pro-apoptotic members (e.g., Bax and Bak) (Ruvolo et al. 2001). The latter then drives cytochrome C release and caspase activation. The apoptotic phenotype consequent to damage is associated with reduced Bad phosphorylation, and down-regulation of Pim1. Pim1 belongs to a family of survival kinases that function downstream of JAK/STAT activation and regulates both cellular apoptosis and metabolism. BTEB2 modulates the apoptotic response in a p53-independent manner and suggest an association between BTEB2 and Bad phosphorylation (Zhao et al. 2008). Pim1 phosphorylates a range of cellular substrates, which includes Bad at two distinct residues: ser112 and ser136 (Aho et al. 2004; Macdonald et al. 2006; Porter and Janicke 1999). Recent data have shown that cell apoptosis can be controlled by a number of survival pathways, which converge to facilitate Bad phosphorylation (Bergmann 2002). This possibility made us to question if BTEB2 functions in the same pathway in PC12 cells. In our studies, active caspase-3 and Bad phosphorylation apparently increase and decrease respectively in PC12 cells hemin induced apoptosis model. As the key factor during execution phase of apoptosis, caspase-3 converge signals from both the intrinsic and extrinsic pathways (Porter and Janicke 1999). Hereby, we speculate that BTEB2 inhibits neuronal apoptosis through Bad phosphorylation pathway after ICH.

BTEB2 regulates both apoptosis in the early phase and proliferation in the late phase, thus indicating that this factor acts as a bimodal and likely central regulator of proliferative cardiovascular pathologies (Suzuki et al. 2009). BTEB2 in some processes are generally mediated by direct transcriptional regulation of its target genes and may have both transactivating and repressive functions. BTEB2 activates the JNK pathway causing apoptosis and reduced cell survival in esophageal squamous cell cancer cells. Transcriptional control of many steps in the JNK pathway by KLF5 is characteristic of a coherent feed-forward loop and is indicative of the critical role of KLF5 in the regulation of this signaling network (Tarapore et al. 2013). The molecular mechanism that BTEB2 regulates apoptosis remains to be explored.

Taken together, our data suggest that BTEB2 prevents neuronal apoptosis after ICH via Bad phosphorylation pathway. Further studies are warranted to inquire the therapeutic potentials of BTEB2 for ICH as well as ischemic stroke and other neurodegenerative diseases.

## Electronic supplementary material

Below is the link to the electronic supplementary material.

Supplement Figure 1 (GIF 53 kb)

High resolution image (TIFF 7670 kb)
