# Supplementary figures and images for: BTEB2 Prevents Neuronal Apoptosis via Promoting Bad Phosphorylation in Rat Intracerebral Hemorrhage Model
Source: J Mol Neurosci. 2014 Apr 27;55(1):206–16. doi: 10.1007/s12031-014-0305-8 (PMC4289975; doi:10.1007/s12031-014-0305-8)

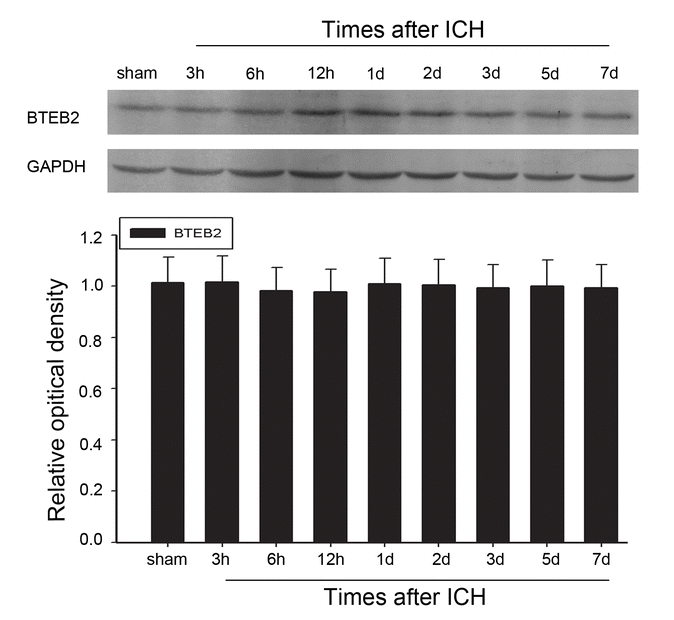

Supplement: Supplementary file 1 — (GIF 53 kb) [file 12031_2014_305_Fig7_ESM.gif]

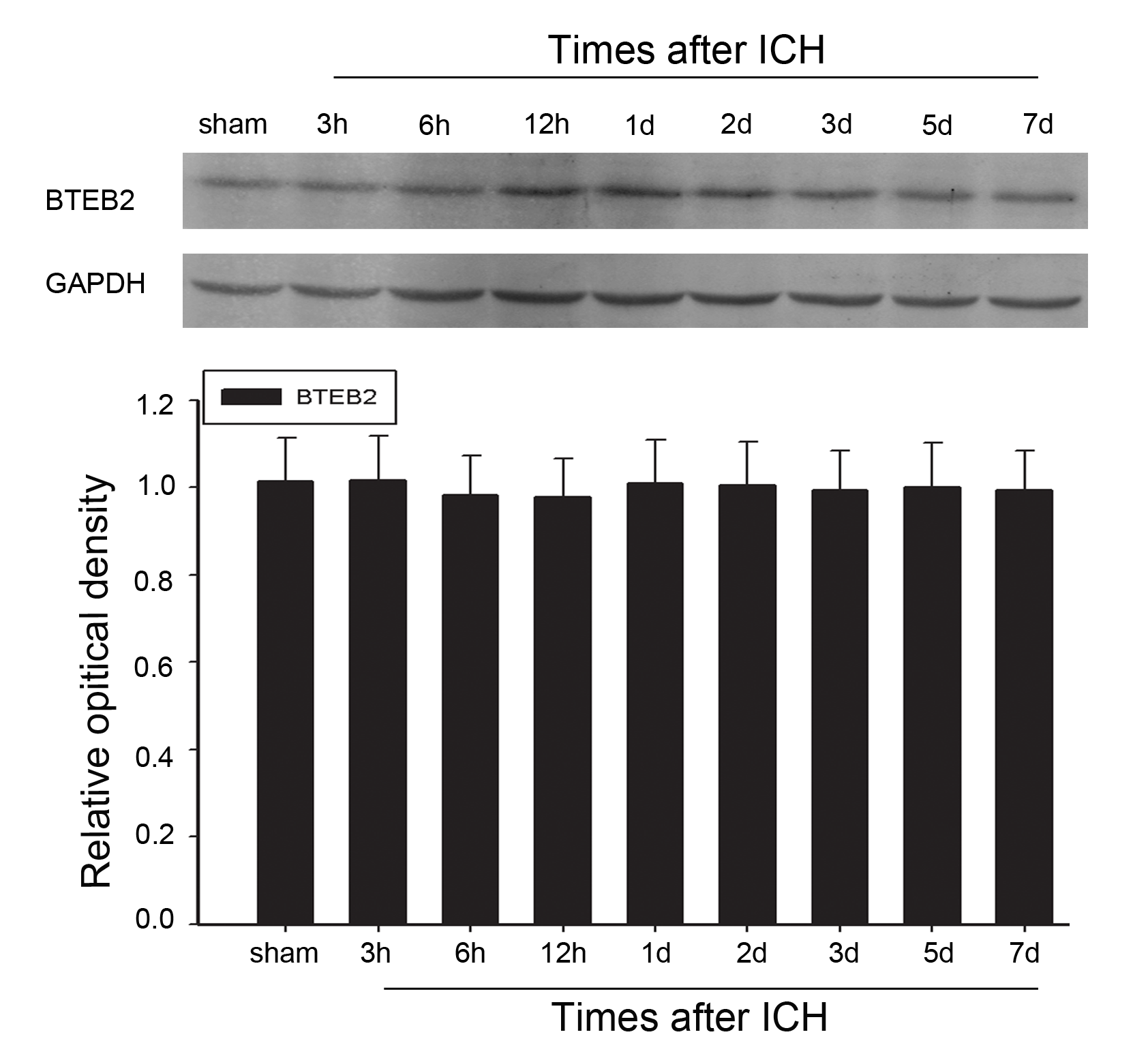

Supplement: Supplementary file 2 — High resolution image (TIFF 7670 kb) [file 12031_2014_305_MOESM1_ESM.tif]
